# Prognostic Value of CRP-Albumin-Lymphocyte (CALLY) Index in Patients Undergoing Surgery for Oral Cavity Cancer

**DOI:** 10.7150/jca.74930

**Published:** 2022-07-27

**Authors:** Yao-Te Tsai, Chien-An Ko, Hung-Chin Chen, Cheng-Ming Hsu, Chia-Hsuan Lai, Yi-Chan Lee, Ming-Shao Tsai, Geng-He Chang, Ethan I. Huang, Ku-Hao Fang

**Affiliations:** 1 Department of Otorhinolaryngology - Head and Neck Surgery, Chang Gung Memorial Hospital, Chiayi, Taiwan.; 2 College of Medicine, Chang Gung University, Taoyuan, Taiwan.; 3 Department of Radiation Oncology, Chang Gung Memorial Hospital, Chiayi, Taiwan.; 4 Department of Otorhinolaryngology - Head and Neck Surgery, Chang Gung Memorial Hospital, Keelung, Taiwan.; 5 Department of Otorhinolaryngology - Head and Neck Surgery, Chang Gung Memorial Hospital, Taoyuan, Taiwan.

**Keywords:** CRP-albumin-lymphocyte index, oral cavity cancer, overall survival, disease-free survival, nomogram

## Abstract

**Background**: The prognostic value of the CRP-albumin-lymphocyte index (CALLY index) was analyzed in patients with oral cavity squamous cell carcinoma (OSCC) undergoing curative surgery.

**Methods:** We retrospectively included 279 patients who were diagnosed as having primary OSCC and being treated with surgery. The optimal cutoff for the preoperative CALLY index was identified by considering the area under the receiver operating characteristic curve; subsequently, the discriminatory ability of the cutoff was determined. We employed Kaplan-Meier analysis and the log-rank test to elucidate associations between the CALLY index and survival outcomes. We identified prognostic variables by using the Cox proportional hazards model. Finally, we devised a nomogram based on the CALLY index for predicting individualized survival.

**Results**: The cutoff value of the CALLY index was determined to be 0.65. A CALLY index < 0.65 exhibited a significant association with pathological aggressiveness as well as shorter overall and disease-free survival (OS and DFS, both *P* < 0.001). A low CALLY index was an independent risk factor for short OS and DFS [hazard ratio = 3.816; 95% confidence interval (CI) 2.393-6.086; *P* < 0.001; and hazard ratio = 2.103; 95% CI 1.451-3.049; *P* < 0.001, respectively] in multivariate Cox analysis. The prognostic nomogram based on the CALLY index yielded accurate predictions of OS, as revealed by a concordance index of 0.797.

**Conclusions:** The preoperative CALLY index is easy and inexpensive to calculate and, in patients with OSCC, can be a valuable prognostic biomarker. The CALLY-index-based nomogram established in this study provides accurate survival predictions.

## Introduction

In 2020, 377,713 new cases of oral cavity cancer were diagnosed, and globally, this type of cancer caused 177,757 deaths 1. Histologically, oral cavity squamous cell carcinoma (OSCC) is the most common type of this cancer 2. In Taiwan, the major OSCC risk factors remain cigarette smoking, alcohol consumption, and betel nut chewing 3. Curative surgery is currently recognized as the mainstay of treatment for OSCC 4, and adjuvant therapy is recommended for those with factors indicating a poor prognosis, such as locally advanced T4 disease 5, poor tumor differentiation 6, perineural invasion (PNI) 7, cervical nodal metastasis 8, or extranodal extension (ENE) 9.

Inflammatory responses in the tumor microenvironment play critical roles in cancer development, tumor progression, and distant metastasis 10. In addition, in patients with cancer, malnutrition is reported to be associated with poor chemotherapy response and low quality of life 11. Therefore, a growing number of studies have identified the high value of using nutrition and inflammation parameters, such as the levels of albumin and serum C-reactive protein (CRP), to predict outcomes in patients with OSCC 12-15. In those with OSCC, serum CRP—an acute inflammation protein produced by the liver—was demonstrated to be a prognostic indicator 13. Albumin is the most abundant protein in human serum 16, and higher all-cause mortality was reported among patients with OSCC with a lower albumin level 12. In addition, lymphocytes are pivotal components of the host anticancer immune response, and pretreatment lymphocyte count was reported to be an independent prognostic factor in human-papillomavirus-related oropharyngeal cancer 14. By integrating the aforementioned parameters, Iida et al. proposed the novel CRP-albumin-lymphocyte (CALLY) index—defined as serum albumin level (g/dL) × absolute lymphocyte count (cells/µL) / CRP level (mg/dL) × 10^4^—and discovered a significant association between low CALLY index and poor overall survival (OS) in those with hepatocellular carcinoma (HCC) after hepatectomy 17. Given the high accessibility and cost-effectiveness of analyzing the inflammation and nutrition-based CALLY index, it may give physicians useful information for prognostication and optimization of treatment planning. Nevertheless, a lack of relevant information on OSCC has precluded the formation of a robust recommendation on use of the CALLY index for OSCC management. In this study, the prognostic value of the CALLY index in patients with surgically treated OSCC was investigated. Given that the CALLY index reflects the host's nutritional status and immuno-inflammation response, we hypothesized that in patients with OSCC, the CALLY index before treatment would be strongly associated with survival outcomes; therefore, the preoperative CALLY index would be useful for identifying patients with an unfavorable prognosis at an early stage. To improve the accuracy of individualized survival predictions and facilitate application of the CALLY index in clinical practice, this study employed the CALLY index to create a prognostic nomogram. The accuracy of OS prediction made using the proposed nomogram model was evaluated using concordance-index (C-index) and calibration plots.

## Materials and methods

### Study design and population

This retrospective study was conducted in accordance with the Declaration of Helsinki and approved by the institutional review board of our hospital (No. 202100017B0C601) prior to commencement of the study. Written informed consent was obtained from all participants. This study recruited 308 consecutive patients with newly diagnosed OSCC at our hospital. The study period was January 2008 to December 2017. The following eligibility criteria were employed: (1) age ≥ 18 years; (2) histopathological diagnosis of primary OSCC; (3) complete pretreatment laboratory data; and (4) underwent curative surgery to treat OSCC at the study hospital. We excluded 29 patients for the following reasons: (1) the presence of unresectable tumor, synchronous cancer, or distant metastasis upon diagnosis (n = 6); (2) neoadjuvant therapy prior to surgery (n = 2); (3) history of cancer or a hematologic disorder (n = 12); (4) evidence of an acute infection or severe inflammatory disease within the 1 month before surgery (n = 2); and (5) missing follow-up data (n = 7). Thus, the final analysis covered 279 patients.

### Data collection

Medical staff reviewed the electronic medical records at our hospital and collected the clinical data of each enrolled patient. Within the 2 weeks prior to surgery, all patients underwent pretreatment workup in accordance with our institution's guidelines; this workup included a detailed physical examination and medical history taking, complete laboratory tests, chest radiography, nuclear bone scanning, head and neck computed tomography (CT) or magnetic resonance imaging (MRI), and ultrasonography of the liver. When a patient was found to have locally advanced disease or suspicious metastatic lesions were identified in the aforementioned examinations, chest and abdominal CT scans and/or a positron emission tomography-CT scan would be arranged to enable precise tumor staging. The value of using clinicopathological features as prognostic factors was carefully reviewed; these features were as follows: sex; age at diagnosis; underlying comorbidities according to the Charlson comorbidity index (CCI) 18; tumor subsite and size; pathological cancer stage, as defined by the American Joint Committee on Cancer eighth-edition cancer staging manual; ENE and PNI status; cancer cell differentiation; depth of invasion (DOI); nearest surgical margin; the need for and types of adjuvant therapy; and personal health-related habits. Cigarette smoking was defined as smoking ≥1 packet of cigarettes daily for ≥1 year; alcohol drinking was defined as consuming ≥2 alcoholic beverages weekly for more than half a year; and betel nut consumption was defined as chewing ≥3 betel nuts daily for ≥1 year 19. Each patient was classified as having no, one, and two or all exposures if they had none, one, and two or more of the aforementioned personal habits, respectively. Documented symptoms and signs and laboratory test results indicated that the enrollees had no active infection.

### Protocol of treatment

The patients' primary OSCC treatment was curative surgery; wide excision of the tumor as well as concurrent unilateral or bilateral neck dissection were employed. Plastic surgeons immediately reconstructed any surgical defects by using local or free flaps. In accordance with the results of an in-house tumor conference and our institution's guidelines, postoperative adjuvant therapy was applied in indicated patients; this therapy was administered within the 6 weeks after surgery. Lin et al. reported our institute's detailed adjuvant therapy guidelines for OSCC 5. Briefly, patients with pathological T4 tumor and/or ipsilateral single nodal metastasis without ENE were administered postoperative radiotherapy (RT) 5. Postoperative chemoradiotherapy (CRT) was offered to patients with a positive surgical margin, multiple nodal metastases, or ENE 5. In the adjuvant intensity-modulated RT, the primary tumor site and high risk areas were subjected to a cumulative radiation dose of 66 Gy; a 2-Gy daily dose of radiation was applied for 5 days per week. In the adjuvant CRT, intravenous cisplatin-based chemotherapy was administered at a weekly dose of 40 mg/m^2^ or triweekly dose of 100 mg/m^2^; the selection between these was made in accordance with the patient's general condition and oncologist's judgment.

### Measurements of serum biomarkers

Serum biomarker-survival outcome associations were evaluated by routinely examining the biochemistry test results and peripheral blood counts in our central laboratory within the 2 weeks before surgery. We used a hematology analyzer (Sysmex SE-9000, Kobe, Japan) to obtain hematologic parameters including hemoglobin level and lymphocyte, neutrophil, and platelet counts. We employed the Cobas 8000 automated biochemistry analyzer (Roche Hitachi, Rotkreuz, Switzerland) to measure preoperative biochemical parameters, namely albumin and CRP (respective reference values: 3.5-5.5 g/dL and <0.5 mg/dL) levels. The CALLY index was determined using the following equation 17:

CALLY index = albumin level (g/dL) × lymphocyte count (/μL) / CRP level (mg/dL) x 10^4^

### Follow-up plan

In the first and second year after surgery, patients were followed up every 2-3 months, with subsequent follow ups every 6 months. During each follow-up visit, a flexible endoscopic examination and a physical examination were routinely performed. Patients underwent MRI or CT every 6 months in the first 2 years after surgery; from the third postoperative year onward, follow-up imaging was performed once every year. We defined OS as the period from the date of curative surgery to that of all-cause mortality, being censored, or final follow-up. We defined disease-free survival (DFS) as the period from the date of curative surgery to that of cancer recurrence, distant metastasis, being censored, or the final follow-up. We considered the follow-up period as the interval between the date of curative surgery and December 31, 2019, or death, whichever occurred first. Survival information was collected from medical chart reviews and telephone interviews.

### Statistical analysis

We report categorical data as numbers and percentages. The Kolmogorov-Smirnov test was employed to assess data normality, and we present normally and nonnormally distributed continuous variables by using the mean and standard deviation and the median and interquartile range, respectively. For continuous and categorical variables, we employed the Mann-Whitney *U* and chi-square tests, respectively, for identifying intergroup differences in clinicopathological features. To identify the optimal cutoff values of serum biomarkers for OS, receiver operating characteristic (ROC) curves subjected to Youden's index correction were obtained; the corresponding area under the ROC curve (AUC) values were also calculated. For the survival analysis, we estimated the OS and DFS by using Kaplan-Meier analysis and determined intergroup survival differences through the log-rank test. We used the Cox proportional hazards model to identify independent DFS and OS risk factors; the hazard ratio (HR) and 95% confidence interval (CI) for each factor are presented. Factors deemed to be significant in the univariate analysis (log-rank test: *p* < 0.1) were used in the multivariable model. The following clinicopathological variables included in the univariate and multivariate analyses (included in addition to the preoperative CALLY index) potentially confounded the prognosis of those with OSCC: sex, age at diagnosis (<65 or ≥65 years), overall stage, PNI (absent or present), cancer cell differentiation [well to moderately differentiated (W-D/M-D) or poorly differentiated (P-D)], nearest surgical margin (≥5 or <5 mm), tumor site (tongue, buccal mucosa, or other), personal habits (no, one, or two or all exposures), need for adjuvant CRT (no or yes), and underlying comorbidities (CCI: 0, 1, or ≥2). We used SPSS 23 (IBM Corp., Armonk, NY, USA) for all statistical analyses, with two-sided *p* < 0.05 indicating a significant finding.

We used the “rms” package in R software (v. 5.1-0; Vanderbilt University, Nashville, TN, USA) to establish a prognostic nomogram incorporating the preoperative CALLY index and clinicopathological features; the endpoints were 3-year and 5-year OS 20. To ascertain the accuracy of OS predictions, we derived the C-index for our nomogram model as well as the conventional American Joint Committee on Cancer (AJCC) staging system. Perfect and random predictability were considered to correspond to a C-index of 1.0 and 0.5, respectively. Moreover, we created calibration plots to assess how consistent the nomogram-derived OS predictions were with the actual survival outcomes.

## Results

### Characteristics of study population

The patients' demographic and clinicopathological characteristics as well as the preoperative laboratory test results are summarized in **Table 1**. Overall, 279 patients with operated OSCC were enrolled, including 249 (89.2%) male patients and 201 (72.0%) patients were under the age of 65 years. The median (interquartile range) age at diagnosis was 56 (52-67) years, with the median (interquartile range) follow-up time being 48.1 (18.7-67.8) months. The tumor site was the tongue in 113 (40.5%) patients and buccal mucosa in 85 (30.5%) patients; these were the two most frequent tumor subsites in this study. Among the 279 patients, 52 (18.6%) had stage I, 38 (13.6%) had stage II, 45 (16.1%) had stage III, and 144 (51.6%) had stage IV OSCC. Regarding regional lymph node involvement, 100 patients (35.8%) had pathologically confirmed cervical nodal metastases, and 55 (19.7%) had ENE. Presence of PNI and a DOI of more than 10 mm were observed in 25.1% (n = 70) and 47.7% (n = 133) of the enrolled patients, respectively. In addition, most patients (n = 248, 88.9%) had W-D/M-D OSCC. Nearly three quarters of patients (n = 206, 73.8%) were reported as having the nearest surgical margin ≥ 5 mm. As for underlying comorbidities, 145 (52.0%) patients had a CCI of 0, 82 (29.4%) patients had a CCI of 1, and 52 (18.6%) patients had a CCI of 2 or greater. Among the included patients, the vast majority smoked (n = 231, 82.8%), chewed betel nuts (n = 222, 79.6%), and drank alcohol (n = 187, 67.0%). All enrolled patients completed the treatment course; 134 (48.2%) patients underwent curative surgery alone, 39 (13.9%) had adjuvant RT, and 106 (37.9%) underwent adjuvant CRT. Prior to surgery, 7 of the 279 (2.5%) patients received a nasogastric feeding tube because of poor oral intake and remarkable malnutrition.

### ROC curve analysis

**Figure 1:**
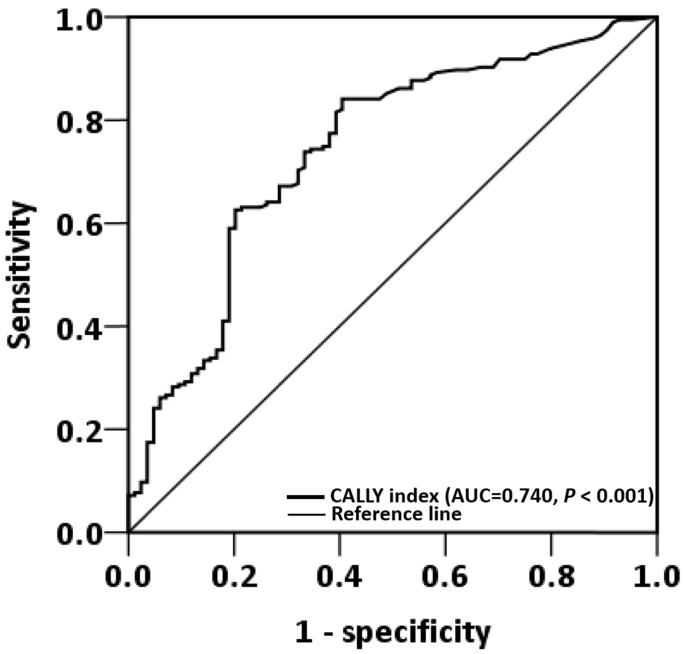
ROC curve analysis of the CALLY index in patients with operated OSCC.

ROC curve analysis revealed that the optimal OS cutoff of the CALLY index was 0.65 (sensitivity, 84.1%; specificity, 60.5%, *P* < 0.001, **Figure 1**). To compare the prognostic discrimination between the CALLY index and its components—serum albumin level, lymphocyte count, and CRP level 13, 21, 22—we performed the ROC curve analysis of these markers and compared the corresponding AUCs. **Table 2** presents these comparisons. The CALLY index was discovered to have a higher AUC (0.740, 95% CI: = 0.676-0.806, *P* < 0.001) than albumin (0.719, 95% CI: = 0.652-0.787, *P* < 0.001), lymphocyte count (0.613, 95% CI: = 0.538-0.687, *P* = 0.003), and CRP (0.708, 95% CI: = 0.641-0.776, *P* < 0.001). These findings suggested that the CALLY index had the optimal prognostic discriminatory ability in this study setting and prompted us to thoroughly assess the prognostic ability of the CALLY index in OSCC.

### Clinicopathological characteristics stratified by CALLY index cutoff

The study population was grouped into two cohorts in accordance with whether they had a CALLY index higher or lower than the cutoff of 0.65; the high- and low-CALLY-index groups comprised 198 (70.9%) and 81 (29.1%) patients, respectively. The distributions of clinicopathological and demographic characteristics in the low-CALLY-index (<0.65) and high-CALLY-index (≥0.65) cohorts are detailed in **Table 3**. Significant correlations were identified between low CALLY index and age ≥ 65 years (*P* = 0.031), stage III or IV disease (*P* = 0.002), cervical nodal metastasis (*P* = 0.003), T3 or T4 classification (*P* < 0.001), DOI ≥ 10 mm (*P* < 0.001), the presence of ENE (*P* < 0.001), the need for adjuvant therapy (*P* = 0.001), and shorter median survival time (*P* < 0.001). However, no significant between-cohort differences were noted terms of in sex (*P* = 0.763), PNI (*P* = 0.415), cancer cell differentiation (*P* = 0.401), surgical margin (*P* = 0.253), tumor subsites (*P* = 0.396), personal habits (*P* = 0.673), or CCI distribution (*P* = 0.091).

### Association between CALLY index and survival outcomes

**Figure 2:**
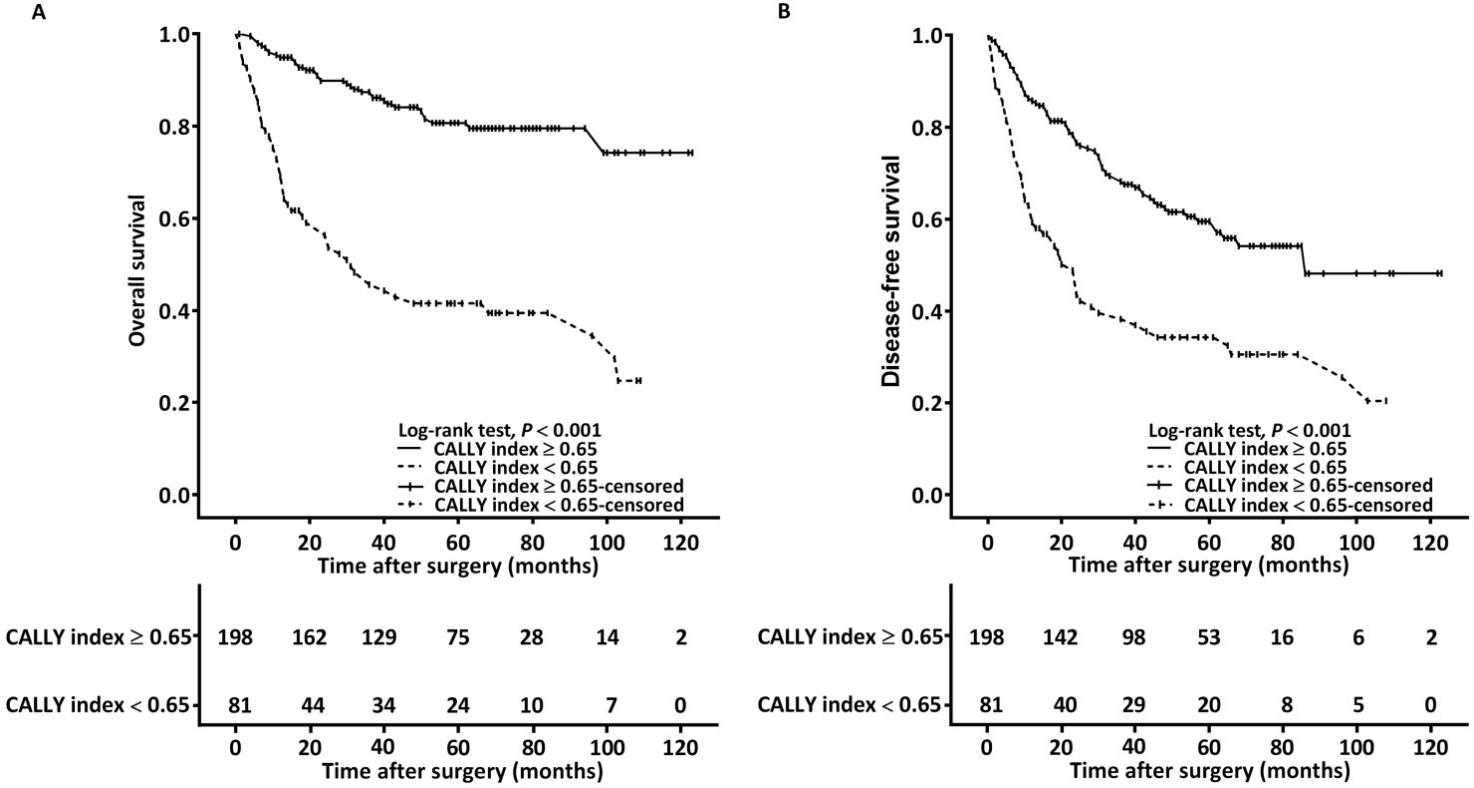
Kaplan-Meier curves displaying the estimated survival probabilities in patients with OSCC, with stratification according to the optimal cutoff value of the CALLY index. **A.** OS analysis.** B.** DFS analysis.

**Figure 3:**
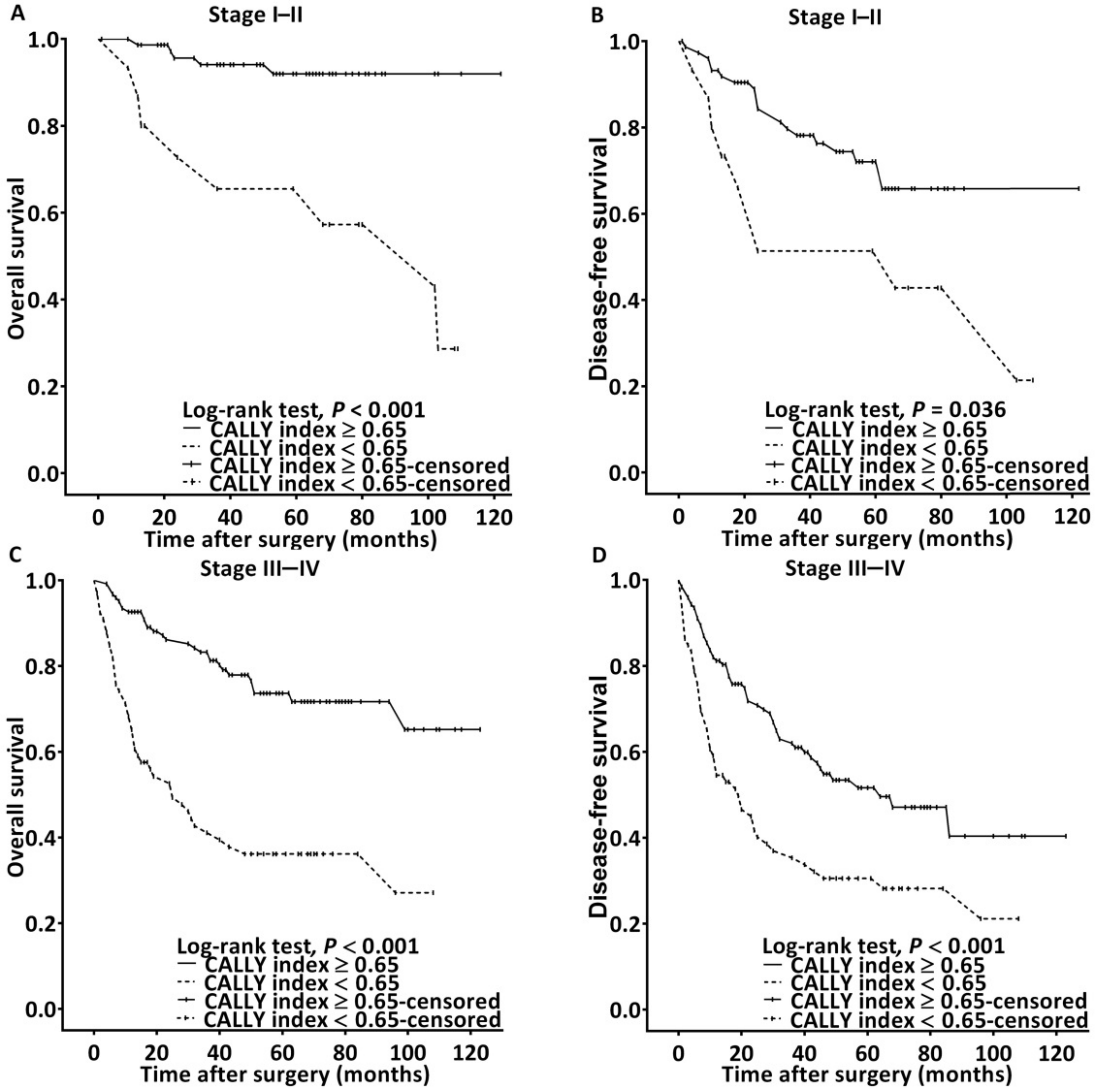
Survival curves for stratification in accordance with the CALLY index cutoff and cancer stage. **A.** OS analysis in stage I-II OSCC.** B.** DFS analysis in stage I-II OSCC **C.** OS analysis in stage III-IV OSCC. **D.** DFS analysis in stage III-IV OSCC.

The Kaplan-Meier survival analysis and log-rank test findings indicated that the estimated median OS was >99 months for the patients with a CALLY index of ≥0.65 and 32 (95% CI 16-46) months for those with a CALLY index of <0.65 (*P* < 0.001, **Figure 2A**). The associations of OS with clinicopathological variables are presented in **Table 4**. In the univariate analysis, poor OS was significantly associated with stage IV disease, PNI, P-D OSCC, surgical margin < 5 mm, need for adjuvant chemotherapy, CCI ≥ 2, and CALLY index < 0.65. Multivariate analysis revealed that for poor OS, the independent risk factors were stage IV disease (HR = 3.811, 95% CI: 1.476-9.839, *P* = 0.006), P-D OSCC (HR = 3.157, 95% CI: 1.686-5.912, *P* < 0.001), CCI ≥ 2 (HR = 1.431, 95% CI: 1.073-3.087, *P* = 0.033), and CALLY index < 0.65 (HR = 3.816, 95% CI: 2.393-6.086, *P* < 0.001). To further elucidate the effect of the CALLY index-cancer stage interaction on OS, we plotted survival curves stratified by CALLY index and cancer stage (**Figure 3**). Regardless of whether the OSCC was in an early or advanced stage, the low-CALLY-index group had a significantly shorter OS compared with the high-CALLY-index group (**Figure 3A** and **3C:** stage I-II and stage III-IV, respectively; both *P* < 0.001).

The estimated median DFS was 86 months for the patients with a CALLY index of ≥ 0.65 and 23 (95% CI 15-31) months for those with a CALLY index of < 0.65 (*p* < 0.001, **Figure 2B**). The univariate analysis findings shown in** Table 5** indicate significant associations of poor DFS with stage IV disease, P-D OSCC, need for adjuvant chemotherapy, and CALLY index < 0.65. Stage IV disease (HR = 2.443, 95% CI: 1.333-4.479, *P* = 0.004), P-D OSCC (HR = 2.560, 95% CI: 1.535-4.269,* P* < 0.001), and CALLY index < 0.65 (HR = 2.103, 95% CI: 1.451-3.049, *P* < 0.001) were independent predictors of poor DFS according to the multivariate analysis (**Table 5**). In addition, the survival curves stratified by CALLY index and cancer stage indicated that, relative to those in the high-CALLY-index group, the patients in the low-CALLY-index group with early (stage I-II, **Figure 3B**) and advanced (stage III-IV, **Figure 3D**) disease had significantly poorer DFS (*P* = 0.036 and < 0.001, respectively).

### Stratified analysis regarding discriminative ability of CALLY index

**Figure 4:**
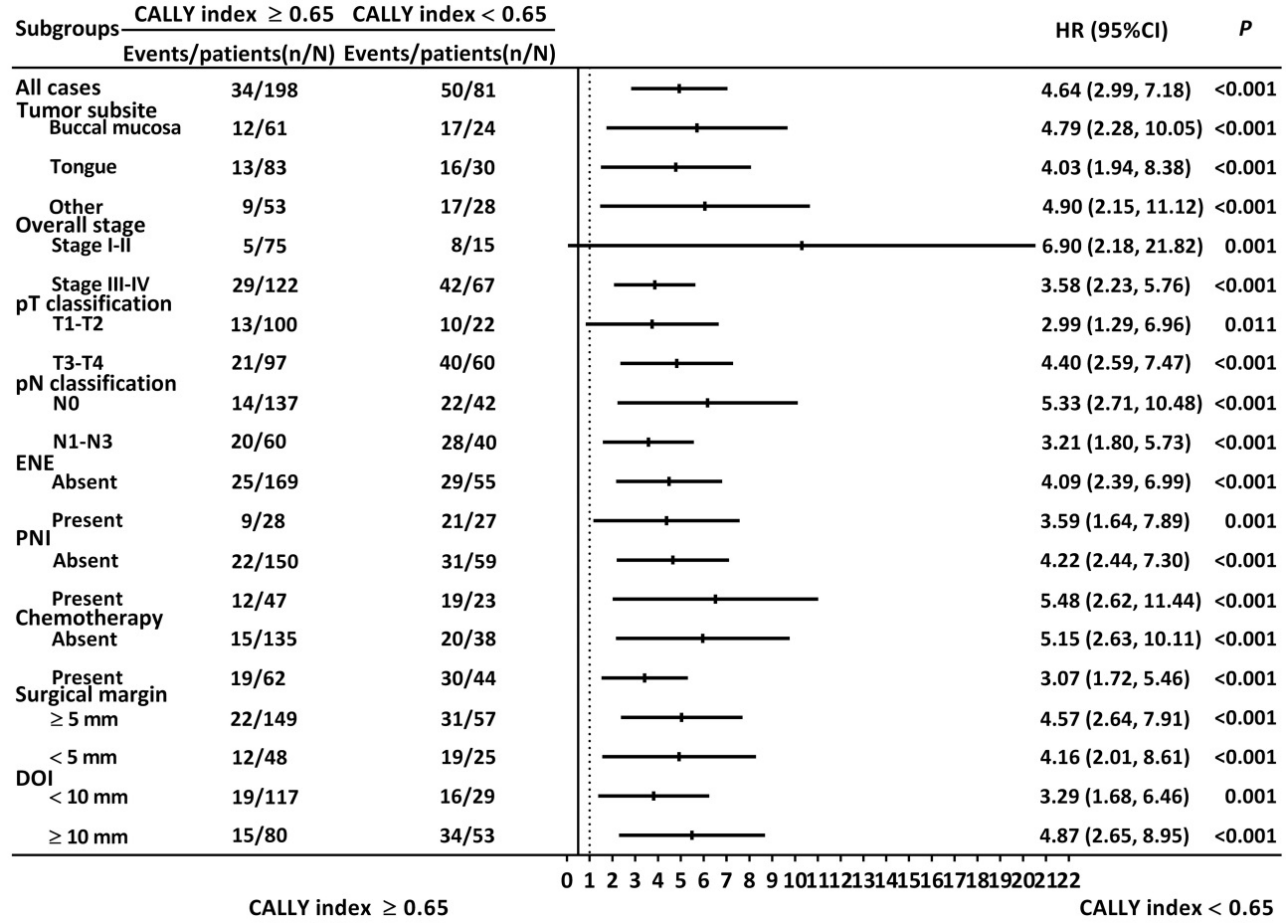
Stratified analysis regarding the discriminative ability of the CALLY index. HR > 1.0 indicated poorer survival.

The results of the stratified analysis are illustrated in **Figure 4**. The CALLY index was consistently associated with OS when patients were grouped by different tumor subsite (buccal cancer: HR: 4.79, 95% CI: 2.28-10.05, *P* < 0.001; tongue cancer: HR: 4.03, 95% CI: 1.94-8.38, *P* < 0.001), tumor-node-metastasis stage (stage I or II: HR: 6.90, 95% CI: 2.18-21.82, *P* = 0.001; stage III or IV: HR: 3.58, 95% CI: 2.23-5.76, *P* < 0.001), T classification (T1 or T2: HR: 2.99, 95% CI: 1.29-6.96, *P* = 0.011; T3 or T4: HR: 4.40, 95% CI: 2.59-7.47, *P* < 0.001), nodal involvement (N0: HR: 5.33, 95% CI: 2.71-10.48, *P* < 0.001; N1-N3: HR: 3.21, 95% CI: 1.80-5.73, *P* < 0.001), ENE status (no ENE: HR: 4.09, 95% CI: 2.39-6.99, *P* < 0.001; with ENE: HR: 3.59, 95% CI: 1.64-7.89, *P* = 0.001), PNI status (no PNI: HR: 4.22, 95% CI: 2.44-7.30, *P* < 0.001; with PNI: HR: 5.48, 95% CI: 2.62-11.44, *P* < 0.001), need for adjuvant chemotherapy (no chemotherapy: HR: 5.15, 95% CI: 2.63-10.11, *P* < 0.001; with chemotherapy HR: 3.07, 95% CI: 1.72-5.46, *P* < 0.001), surgical margin (margin ≥ 5 mm: HR: 4.57, 95% CI: 2.64-7.91, *P* < 0.001; margin < 5 mm: HR: 4.16, 95% CI: 2.01-8.61, *P* < 0.001), and DOI status (DOI ≥ 10 mm: HR: 4.87, 95% CI: 2.65-8.95, *P* < 0.001; DOI < 10 mm: HR: 3.29, 95% CI: 1.68-6.46, *P* = 0.001).

### Nomograms for OS prediction

**Figure 5:**
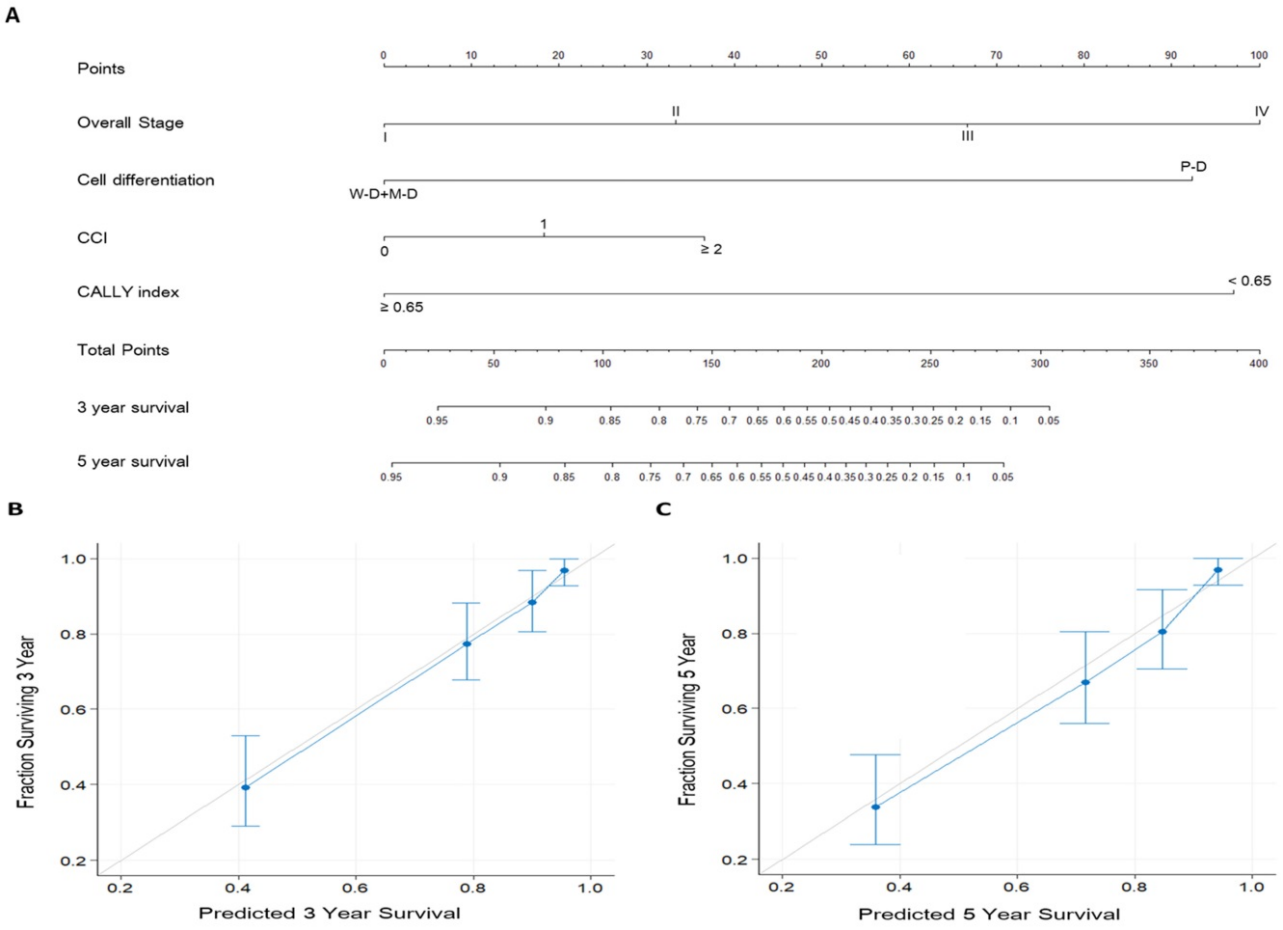
Prognostic nomogram established for prediction of 3-year and 5-year OS. **A.** Nomogram incorporating the CALLY index and clinicopathological factors. Each factor is connected to the uppermost point score by a vertical line, and the corresponding points represent how much this factor added to the risk. A total points score is obtained by summing the points from each factor and converted into 3-year and 5-year OS probabilities by drawing vertical lines to the respective axis below. **B.** Calibration plots of 3-year OS. **C.** Calibration plots of 5-year OS. Calibration plots indicate the consistency between nomogram-predicted survival and actual outcomes. Perfect survival prediction is indicated by the 45° light-gray line. The nomogram performance and 95% CIs for survival are indicated blue dots and bars, respectively.

To enhance the accuracy of individualized OS prediction in patients with OSCC, we created a prognostic nomogram on the basis of the independent prognostic factors identified in the multivariate analysis—overall stage, cancer cell differentiation, CCI, and CALLY index **(Figure 5)**. For comparison, we also established a nomogram on the basis of AJCC stage alone. **Figure 5A** depicts the proposed nomogram for estimating the 3-year and 5-year OS; the corresponding AUC was 0.81 (sensitivity, 81.1%; specificity, 71.3%). The nomogram incorporating CALLY index and clinicopathological factors had a C-index (95% CI) of 0.797 (0.760-0.826); for the nomogram incorporating AJCC stage only, the C-index was 0.672 (0.641-0.703). **Figure 5B** and **5C** presents the calibration plots of 3-year and 5-year OS probabilities, respectively, estimated using the proposed nomogram. The plots show high consistency with the 45° diagonal, which indicates good prediction. The nomogram model was thus concluded to have high calibration accuracy. All these results suggest the informative role of the CALLY index in OSCC and indicate that the nomogram incorporating the CALLY index has high performance in estimating the OS of patients with OSCC after surgery.

## Discussion

Currently, the AJCC staging system integrating tumor extent, regional lymph node status, and distant metastasis status is the tool most frequently used in the OSCC context for optimizing treatments, predicting survival, and stratifying patient groups. A limitation of this system is that it accounts only for cancer characteristics; it neglects host factors that can affect oncologic outcomes, such as patients' nutrition and systemic inflammatory status 23. Evidence is increasingly showing that cancer development is closely associated with the host's nutrition and the inflammatory response within the tumor microenvironment 24, 25 and that some of these nutrition and inflammation-related parameters—such as serum albumin and CRP levels and lymphocyte count—have the potential to predict OSCC prognosis 12-14. Iida et al. proposed the CALLY index, which combines indicators of the host's nutrition (serum albumin level) and immune-inflammatory response (serum CRP level and lymphocyte count), and demonstrated its superior prognostic discrimination in patients with HCC after hepatectomy 17. However, relevant information on the CALLY index in OSCC has been lacking, and whether this index yields prognostic discrimination superior to that of its components (albumin and CRP levels and lymphocyte count) is also uncertain for OSCC. Our review of the literature suggests that this study is the first to explore the prognostic value of the CALLY index in the context of OSCC treated with curative surgery. The ROC curve analysis revealed that the AUC of the CALLY index was higher than that of albumin level, CRP level, and lymphocyte count, suggesting that the CALLY index has better prognostic discrimination for OS than its component elements. In addition, significant associations of a low CALLY index (<0.65) with older age (≥65 years), adverse clinicopathological characteristics (e.g. stage III or IV disease, late pT classification, cervical nodal metastasis, DOI ≥ 10 mm, and presence of ENE), need for adjuvant therapy, and relatively short median survival were discovered, suggesting the importance of a survey for malnutrition and inflammatory status before the operation with regard to OSCC prognosis and disease aggressiveness. Patients with a preoperative CALLY index of <0.65 exhibited significantly shorter median OS and DFS than those with a CALLY index ≥ 0.65, as indicated by the log-rank and Kaplan-Meier results. Our multivariate Cox analyses revealed that a low CALLY index (<0.65) is an independent risk factor for all-cause mortality and treatment failure in OSCC, increasing the corresponding risks by factors of 3.816 and 2.103, respectively, when adjusting for various confounding factors. In addition, the CALLY index exhibited consistent prognostic performance for OS across different subgroups. Overall, our hypothesis was confirmed, suggesting that in patients with operated OSCC, the CALLY index before surgery could be a promising prognostic biomarker. Extending from these findings, patient who has a lower CALLY index before surgery may have poor survival outcome due to the unfavorable general condition for conventional curative treatment. A more careful and personalized therapeutic strategy, as well as follow-up in a shorter interval, may be necessary for these patients.

Although this study's results support a prognostic role of the CALLY index in OSCC, the precise mechanisms underlying the associations of low CALLY index with adverse clinical and survival outcomes in those with OSCC remain uncertain. Our results have revealed that a low CALLY index (<0.65) is associated with adverse pathological features and may be indicative of poor nutrition 26 and low antitumor immunity 27, increased systemic inflammatory response 28, or both, and these findings may provide insights into the prognostic mechanism of the CALLY index in OSCC. In clinical settings, serum albumin is frequently used as a marker to reflect a person's nutritional status 26, and malnutrition and cachexia are common and critical problems in patients with OSCC 29. Serum albumin level was also demonstrated by Bao et al. in their prospective study to be inversely associated with OS among 1395 patients with OSCC 12. Tsai et al. assessed the albumin level of 233 patients who had locally advanced head and neck cancer at two time points, before and after curative surgery, and demonstrated significant correlations of lower preoperative albumin level with adverse OS, DFS, and disease-specific survival 22. Cancer-induced reprogramming of the host's glucose metabolism may cause insulin resistance and further decrease the serum albumin production 30. In addition, tumor-cell-derived proinflammatory cytokines such as interleukin-6 (IL-6) and tumor necrosis factor alpha in turn reduce the hepatic synthesis of albumin 31. This evidence suggests that cancer-associated malnutrition and cachexia may be accompanied by hypoalbuminemia, skeletal muscle wasting, low physical activity, and poor quality of life, which ultimately contribute to the poor survival outcomes in OSCC 12, 32, 33. The peripheral lymphocyte count is another element of the CALLY index, and lymphocytes play an essential role in initiating and activating the adaptive immune response 34. CD4+ T helper cells produce cytokines such as IL-2 and interferon gamma and promote activation and recruitment of CD8+ cytotoxic T cells 35, which exert antitumor effects and directly cause cancer cell destruction through the release of perforin and granzyme 36. Given that the peripheral lymphocyte count reflects a person's cytotoxic immune function 37, the presence of more T helper and tumor-infiltrating cytotoxic T cells was found to correlate with better prognosis for solid tumors 38. However, in the tumor environment, upregulated programmed death ligand-1 may constrain the antitumor response of cytotoxic T cells and reduce the proliferation of tumor-infiltrating lymphocytes 39, 40. In addition, proapoptotic ligands such as Fas ligand and tumor necrosis factor-beta were reported to be produced in patients with cancer and promote the apoptosis of lymphocytes 41. Therefore, lymphopenia was reported to be associated with increased chemotherapy toxicity and poor OS in many malignancies 42, including head and neck cancer 14. Kreinbrink et al. assessed the pretreatment absolute lymphocyte count of 201 patients who underwent radiotherapy for oropharyngeal cancer and demonstrated that pretreatment lymphocyte count could independently predict survival 14. Finally, serum CRP level is the denominator of the CALLY index; it is an acute-phase protein that increases following IL-6 secretion by T cells and macrophages and reflects the systemic inflammatory response 43. The proinflammatory cytokine IL-6 is commonly upregulated in various types of cancer including OSCC 44, and the elevated IL-6 level may promote nodal and distant metastasis by activating the PI3K/AKT/mTOR signaling pathway and correlate with cancer proliferation and poor survival 45. CRP elevation in response to IL-6 upregulation may be a mechanism linking high serum CRP level and poor prognosis. Regarding the tumor microenvironment, as the volume of a tumor increases, inadequate supply of blood to the tumor could result in central necrosis and inflammation of the tumor 46, and the direct extension and penetration of cancer cells could also lead to inflammation and tissue damage such as bone destruction 47. Therefore, elevated CRP may not only be a response to progression of the tumor burden but also reflect tumor lysis and local tissue damage, which ultimately correlates with adverse survival outcomes 48. The aforementioned studies have provided evidence of the mechanism through which a low CALLY index may have an adverse effect on prognosis in those with OSCC; nonetheless, the exact mechanism should be further explored.

The widely used AJCC staging system considers only tumor characteristics. However, patient factors such as antitumor immunity, systemic inflammation, and nutritional status are also significantly associated with the survival outcomes of OSCC 12, 13, 19, and not considering these factors when prognosticating may impair the accuracy of survival predictions. Given that CALLY index integrates the peripheral lymphocyte count with serum CRP and albumin levels in an easily calculable manner, we developed a prognostic nomogram incorporating the CALLY index, AJCC stage system, and clinicopathological features to provide individualized 3-year and 5-year OS estimations and favorable results were obtained (C-index: 0.797; AUC: 0.81). The calibration plots also indicated that the OS probabilities predicted using the proposed nomogram and the actual survival outcomes were in good consistency. All the aforementioned results verify the high performance of the proposed nomogram and suggest the informative role of the CALLY index in patients with OSCC, possibly aiding in personalized treatment planning and therefore affecting all aspects of cancer management; nevertheless, further research is warranted.

Notably, the method used to determine the cutoff value, different primary tumor sites and cancer stages, and a diverse age distribution in a study cohort may all affect the cutoff value of a prognostic indicator. In the current study setting, the most applicable cutoff value of the CALLY index was identified as 0.65 for OSCC prognostication, whereas Iida et al. selected an optimal cutoff value of 5 by using ROC curve analysis in patients with HCC 17. By examining the laboratory data of patients enrolled in our study and Iida et al., we found that our OSCC cohort had a 3.6-fold higher median serum CRP level compared with that of patients with HCC reported by Iida et al. (0.36 vs. 0.10 mg/dL) 17, which can be partially explained by increased oral and systemic inflammation resulting from oral microbiota 49. In addition, patients with OSCC are susceptible to malnutrition 50 and food intake dysfunction 51 and therefore have a decreased serum albumin level. All these factors may contribute to the lower cutoff value for the CALLY index in our study than the cutoff reported for the HCC cohort. Given that the optimal cutoff value of the CALLY index is likely to vary between different cancer sites, this aspect warrants further investigation.

Some of the strengths of this study are as follows: it is the first exploration of the prognostic role of the CALLY index in OSCC, the patient homogeneity in our relatively large cohort was high, and a prognostic nomogram was established that can be used to realize the clinical application of CALLY index and predict OS accurately. We also analyzed various clinicopathological factors and compared low- and high-CALLY-index groups to eliminate the effects of potential confounders. Because the CALLY index is inexpensive, convenient, and simple to calculate from the results of routine laboratory tests and has high prognostic accuracy, it is likely to have high utility in daily clinical practice. Future research on the CALLY index should explore its prognostic role in terms of whether it is affected by treatment modality and whether the benefits of a therapy can be assessed by evaluating its posttreatment versus pretreatment values. Some limitations of our study should be considered. First, it was conducted in a single center and retrospectively analyzed data in an institutional registry, which inevitably causes a certain bias. To reduce the potential bias, we enrolled a relatively large and homogenously treated OSCC cohort. Second, before an operation, the CALLY index may be influenced by subtle factors including indolent infection, undetected liver disease, and an aging-related physiological decrease in serum albumin level 52. In addition, validation of our results with an independent dataset is necessary; external validation might strengthen the evidence supporting the prognostic value of the CALLY index in OSCC. All in all, our results should be validated through further large-scale, multi-institutional, prospective studies before the CALLY index can be recommended for general clinical use.

## Conclusion

This is the first study to show that the preoperative CALLY index can be a promising prognostic biomarker in those who have undergone curative surgery for OSCC. The established nomogram integrating the CALLY index and clinicopathological factors yields accurate OS predictions that can aid individualized prognostication and treatment planning. Given the high cost-effectiveness and availability of the CALLY index, we believe it can be a feasible biomarker for OSCC management and cancer research.

## Figures and Tables

**Table 1:** Baseline characteristics of study participants

Variable	Characteristics
**Age (years)**	
< 65	201 (72.0%)
≥ 65	78 (28.0%)
**Sex**	
Men	249 (89.2%)
Women	30 (10.8%)
**Site of the primary tumor**	
Tongue	113 (40.5%)
Buccal mucosa	85 (30.5%)
Gingiva	34 (12.2%)
Retromolar trigone	18 (6.5%)
Mouth floor	13 (4.7%)
Lip	11 (3.9%)
Hard palate	5 (1.7%)
**AJCC stage**	
I	52 (18.6%)
II	38 (13.6%)
III	45 (16.1%)
IV	144 (51.7%)
**Tumor size (T classification)**	
T1	70 (25.1%)
T2	52 (18.6%)
T3	44 (15.8%)
T4	113 (40.5%)
**Nodal metastasis (N classification)**	
N0	179 (64.2%)
N1	30 (10.8%)
N2	55 (19.7%)
N3	15 (5.3%)
**PNI**	70 (25.1%)
**ENE**	55 (19.7%)
**Cancer cell differentiation**	
W-D/M-D	248 (88.9%)
P-D	31 (11.1%)
**Surgical margin**	
≥ 5 mm	206 (73.8%)
< 5 mm	73 (26.2%)
**DOI ≥ 10 mm**	133 (47.7%)
**CCI**	
0	145 (52.0%)
1	82 (29.4%)
≥ 2	52 (18.6%)
**Personal Habits**	
Cigarette Smoking	231 (82.8%)
Betel nut chewing	222 (79.6%)
Alcohol consumption	187 (67.0%)
**Treatment**	
Surgery only	134 (48.0%)
Surgery + RT	39 (14.0%)
Surgery + CRT	106 (38.0%)
**Laboratory results**	
WBC (X10^3^/μL), median (IQR)	7.70 (6.30-9.70)
Neutrophil (X10^3^/μL), median (IQR)	4.80 (3.59-6.44)
Lymphocyte (X10^3^/μL), median (IQR)	2.01 (1.58-2.59)
CRP (mg/dL), median (IQR)	0.36 (0.13-1.52)
Albumin (g/dL), median (IQR)	4.43 (4.10-4.68)
CALLY index, median (IQR)	2.51 (0.52-7.45)

**Table 2:** Comparison of the AUC values of CALLY index and its components

	AUC	95% CI	*P*	*P*
Nutrition and inflammatory markers				
Albumin	0.719	(0.652-0.787)	<0.001	<0.001
Lymphocyte	0.613	(0.538-0.687)	0.003	0.001
CRP	0.708	(0.641-0.776)	<0.001	<0.001
CALLY index	0.740	(0.676-0.806)	<0.001	-

^a^The AUC values between the CALLY index and other markers were compared using the Z test.

**Table 3:** Clinicopathological characteristics based on the cut-off of CALLY index

Variable	CALLY index ≥ 0.65 (n = 198)	CALLY index < 0.65 (n =81)	*P*
**Sex**			0.763^a^
Men	176 (70.7%)	73 (29.3%)	
Women	22 (73.3%)	8 (26.7%)	
**Age**			0.031^a^
< 65	150 (74.6%)	51 (25.4%)	
≥ 65	48 (61.5%)	30 (38.5%)	
**AJCC stage**			0.002^a^
I-II	75 (83.3%)	15 (16.7%)	
III-IV	123 (65.0%)	66 (35.0%)	
**Tumor size (T classification)**			<0.001^a^
T1-T2	100 (82.0%)	22 (18.0%)	
T3-T4	98 (62.4%)	59 (37.6%)	
**Nodal metastasis (N classification)**			0.003^a^
N0	138 (77.1%)	41 (22.9%)	
N1-N3	60 (60.0%)	40 (40.0%)	
**PNI**			0.415^a^
Absent	151 (72.2%)	58 (27.8%)	
Present	47 (67.1%)	23 (32.9%)	
**ENE**			<0.001^a^
Absent	170 (75.9%)	54 (24.1%)	
Present	28 (50.9%)	27 (49.1%)	
**Cancer cell differentiation**			0.401^a^
W-D/M-D	178 (71.8%)	70 (28.2%)	
P-D	20 (64.5%)	11 (35.5%)	
**Surgical margin**			0.253^a^
≥ 5 mm	150 (72.8%)	56 (27.2%)	
< 5 mm	48 (65.8%)	25 (34.2%)	
**DOI ≥ 10 mm**			<0.001^a^
No	117 (80.1%)	29 (19.9%)	
Yes	81 (60.9%)	52 (39.1%)	
**Tumor sites**			0.396^a^
Tongue	84 (74.3%)	29 (25.7%)	
Buccal mucosa	61 (71.8%)	24 (28.2%)	
Other	53 (65.4%)	28 (34.6%)	
**Personal habits**			0.673^a^
No exposure	24 (75.0%)	8 (25.0%)	
One exposure	14 (77.8%)	4 (22.2%)	
Two or all exposure	160 (69.9%)	69 (30.1%)	
**Treatment**			0.001^a^
Surgery only	103 (76.9%)	31 (23.1%)	
Surgery + RT	33 (84.6%)	6 (15.4%)	
Surgery + CRT	62 (58.5%)	44 (41.5%)	
**CCI**			0.091^a^
0	111 (76.6%)	34 (23.4%)	
1	52 (63.4%)	30 (36.6%)	
≥ 2	35 (67.3%)	17 (32.7%)	
**Laboratory results**			
Albumin(g/dL), median (IQR)	4.55 (4.32-4.72)	4.06 (3.59-4.41)	<0.001^b^
WBC (X10^3^/μL), median (IQR)	7.45 (6.18-9.20)	8.20 (6.55-10.85)	0.055^b^
Neutrophil (X10^3^/μL), median (IQR)	4.54 (3.58-6.03)	5.48 (3.70-7.60)	0.007^b^
Lymphocyte (X10^3^/μL), median (IQR)	2.09 (1.66-2.65)	1.87 (1.43-2.34)	0.001^b^
CRP (mg/dL), median (IQR)	1.85 (0.92-4.16)	3.65 (1.88-7.18)	<0.001^b^
Survival in months, median (IQR)	50.00 (31.75-70.00)	26.50 (10.75-66.50)	<0.001^b^

^a^the Chi-square test. *^b^the Mann-Whitney U test.*

**Table 4:** Multivariate analysis of OS in patients with oral cavity squamous cell carcinoma

Variable		Univariate analysis		Multivariate analysis	
	Survival	HR (95% CI)	*P*	HR (95% CI)	*P*
**Sex**					
Women	76.1%	Reference		Reference	
Men	68.1%	1.541 (0.709-3.351)	0.275	0.997 (0.443-2.243)	0.995
**Age (years)**					
< 65	70.2%	Reference		Reference	
≥ 65	65.7%	1.261 (0.801-1.986)	0.316	1.037 (0.619-1.737)	0.890
**AJCC stage**					
I	91.6%	Reference		Reference	
II	81.5%	1.653 (0.555-4.919)	0.367	2.159 (0.712-6.544)	0.174
III	85.0%	1.331 (0.446-3.972)	0.608	1.354 (0.449-4.085)	0.590
IV	52.7%	5.157 (2.228-11.935)	<0.001	3.811 (1.476-9.839)	0.006
**PNI**					
Absent	74.3%	Reference		Reference	
Present	52.9%	2.178 (1.395-3.398)	0.001	1.443 (0.854-2.439)	0.171
**Cell differentiation**					
W-D/M-D	72.8%	Reference		Reference	
P-D	39.7%	3.396 (2.000-5.768)	<0.001	3.157 (1.686-5.912)	<0.001
**Surgical margin**					
≥ 5 mm	72.9%	Reference		Reference	
< 5mm	58.6%	1.703 (1.091-2.658)	0.019	1.319 (0.806-2.159)	0.271
**Tumor sites**					
Tongue	72.0%	Reference			
Buccal mucosa	65.7%	1.295 (0.774-2.168)	0.325		
Other	68.8%	1.188 (0.700-2.018)	0.523		
**Personal habits**					
No exposure	75.3%	Reference			
One exposure	53.4%	2.245 (0.814-6.193)	0.118		
Two or all exposure	69.4%	1.439 (0.661-3.134)	0.359		
**Adjuvant chemotherapy**					
No	79.4%	Reference		Reference	
Yes	52.1%	2.907 (1.881-4.492)	<0.001	1.388 (0.724-2.162)	0.570
**CCI**					
0	74.9%	Reference		Reference	
1	66.4%	1.457 (0.877-2.423)	0.146	1.228 (0.705-2.136)	0.468
≥ 2	57.9%	1.965 (1.157-3.335)	0.012	1.431 (1.073-3.087)	0.033
**CALLY index**					
≥ 0.65	80.6%	Reference		Reference	
< 0.65	42.2%	4.544 (2.934-7.037)	<0.001	3.816 (2.393-6.086)	<0.001

**Table 5:** Multivariate analysis of DFS in patients with oral cavity squamous cell carcinoma

Variable		Univariate analysis		Multivariate analysis	
	Survival	HR (95% CI)	*P*	HR (95% CI)	*P*
**Sex**					
Women	69.7%	Reference		Reference	
Men	49.8%	1.613 (0.864-3.011)	0.133	1.180 (0.625-2.225)	0.610
**Age (years)**					
< 65	51.5%	Reference		Reference	
≥ 65	53.3%	0.914 (0.622-1.343)	0.647	0.884 (0.594-1.318)	0.546
**AJCC stage**					
I	70.5%	Reference		Reference	
II	66.7%	0.937 (0.447-1.964)	0.864	1.072 (0.508-2.261)	0.855
III	65.9%	1.002 (0.499-2.013)	0.994	1.114 (0.550-2.256)	0.764
IV	37.6%	2.445 (1.452-4.118)	0.001	2.443 (1.333-4.479)	0.004
**PNI**					
Absent	55.0%	Reference			
Present	43.1%	1.355 (0.922-1.991)	0.122		
**Cell differentiation**					
W-D/M-D	55.1%	Reference		Reference	
P-D	30.5%	2.383 (1.487-3.819)	<0.001	2.560 (1.535-4.269)	<0.001
**Surgical margin**					
≥ 5 mm	54.8%	Reference			
< 5mm	44.9%	1.369 (0.947-1.978)	0.095		
**Tumor sites**					
Tongue	57.8%	Reference			
Buccal mucosa	48.1%	1.203 (0.789-1.834)	0.390		
Other	48.2%	1.349 (0.890-2.045)	0.158		
**Personal habits**					
No exposure	70.3%	Reference			
One exposure	41.5%	2.067 (0.859-4.972)	0.105		
Two or all exposure	50.6%	1.744 (0.911-3.338)	0.093		
**Adjuvant chemotherapy**					
No	58.9%	Reference		Reference	
Yes	41.1%	1.761 (1.247-2.486)	0.001	1.369 (0.687-2.415)	0.260
**CCI**					
0	53.2%	Reference			
1	51.6%	1.083 (0.724-1.621)	0.698		
≥2	50.2%	1.127 (0.719-1.765)	0.603		
**CALLY index**					
≥ 0.65	59.3%	Reference		Reference	
< 0.65	35.0%	2.260 (1.594-3.203)	<0.001	2.103 (1.451-3.049)	<0 0.001

## Abbreviations

AJCC: American Joint Committee on Cancer
AUC: area under the receiver operating characteristic curve
C-index: concordance-index
CALLY index: C-reactive protein-albumin-lymphocyte index
CCI: Charlson comorbidity index
CI: confidence interval
CRP: C-reactive protein
CRT: chemoradiotherapy
CT: computed tomography
DFS: disease-free survival
DOI: depth of invasion
ENE: extranodal extension
HCC: hepatocellular carcinoma
HR: hazard ratio
IL: interleukin
IQR: interquartile range
M-D: moderately differentiated squamous cell carcinoma
MRI: magnetic resonance imaging
OS: overall survival
OSCC: oral cavity squamous cell carcinoma
P-D: poorly differentiated squamous cell carcinoma
PNI: perineural invasion
ROC: receiver operating characteristic
WBC: white blood cell
W-D: well-differentiated squamous cell carcinoma
